# Occupational contact allergy in bricklayers, tile setters etc. - Current spectrum of sensitization and recent time trends 

**DOI:** 10.5414/ALX01593E

**Published:** 2017-08-04

**Authors:** J. Geier, H. Lessmann, C. Skudlik, B.K. Ballmer-Weber, E. Weisshaar, W. Uter, A. Schnuch

**Affiliations:** 1 Informationsverbund Dermatologischer Kliniken (IVDK), Institut an der Universität Göttingen, Germany,; 2 Abteilung Dermatologie, Umweltmedizin und Gesundheitstheorie, Universität Osnabrück und Institut für interdisziplinäre dermatologische Prävention und Rehabilitation (iDerm) an der Universität Osnabrück, Germany,; 3 Dermatologische Klinik, Universitätsspital Zürich, Switzerland,; 4 Abteilung Klinische Sozialmedizin, Universitätsklinikum Heidelberg, Germany,; 5 Institut für Medizininformatik, Biometrie und Epidemiologie (IMBE) der Universität Erlangen-Nürnberg, Erlangen, Germany

**Keywords:** building trade, bricklayers, tile setters, occupational dermatitis, contact allergy, chromate, epoxy resin, thiurams

## Abstract

Background: Occupational exposure of bricklayers, construction workers, tile setters etc. has changed during the last years. For some years now, all manually handled cement in the European Union and in Switzerland is chromate-reduced. Epoxy resin systems are being used in more and more fields of application. Improved worker’s protection, especially wearing protective gloves, is promoted. These changes influence the spectrum of occupational contact sensitization. Objective: Description of the current allergen spectrum in patients working in the building trade who suffer from occupational contact dermatitis. Material and methods: Retrospective analysis of data of the Information Network of Departments of Dermatology (IVDK), 2009 – 2011. Results: During the study period, 245 bricklayers, construction workers, tile setters etc. with occupational dermatitis have been patch tested. Potassium dichromate was the most frequent allergen, yielding 15.1% positive reactions, followed by epoxy resin with 13.7% positive reactions. Beyond that, there were 8 additional components of epoxy resin systems (5 reactive diluents and 3 amine hardeners), as well as 9 rubber ingredients, mainly thiurams, among the 30 most frequent allergens. In the course of time, a decline of chromate sensitization could be noted, paralleled by a decline of cobalt sensitization. In contrast, sensitization to epoxy resin has increased. Conclusion: Thanks to the usage of chromate-reduced cement, chromate sensitization continues to decline in the building trade. The increase of epoxy resin sensitization must prompt intensified prevention efforts. When recommending protective gloves, thiuram-free products should be preferred. The most important allergens are covered by the following test series recommended by the German Contact Dermatitis Research Group (DKG): DKG baseline series, DKG test series “building trade”, DKG rubber series.

German version published in Dermatologie in Beruf und Umwelt, Vol. 60, No. 4/2012, pp. 136-150

## Introduction 

Occupational exposure of bricklayers, construction workers, tile setters, and workers in similar professions has changed significantly over the past years. Since the year 2000, all manually-handled cement has to be chromate-reduced in Germany, according to an industry agreement [14]. In July 2003, the European Union (EU) stipulated that chromate-reduced cement has to be used wherever skin contact is possible [2]. This regulation had to be implemented by all EU member states by January 2005 at the latest [2, 28]. Switzerland introduced similar regulations in July 2007 [3]. Thus, exposure to chromate due to contact with cement or cement-containing substances has since been reduced. On the other hand, exposure to epoxy resin systems has been increasing in the building trade [13]. Epoxy resins are no longer exclusively used for heavily-used industrial flooring systems or special coatings, for concrete sealing, or for concrete repair, but also for a number of other purposes, particularly for the (also decorative) coating of floors that are not so heavily used. The German employer’s liability insurance association of the building trade (Berufsgenossenschaft der Bauwirtschaft – BG BAU), together with manufacturers of construction chemicals and other related parties, are trying to improve health and safety in the building trade, particularly in the handling of epoxy resin systems [34]. Among other things, the use of adequate gloves and skin protectants is recommended. All these factors lead to a change in the occupational exposure to potential allergens. Based on data collected by the Information Network of Departments of Dermatology (Informationsverbund Dermatologischer Kliniken –IVDK), we will describe the current spectrum of occupational sensitization as well as the most important changes observed over the past years. 

## Material and methods 

This retrospective analysis is based on data collected by IVDK: clinical data, data on patient history, and test results derived from the examination of patients with occupational dermatitis who worked in the professional group “bricklayers and similar, manufacturer of construction material” at the time of examination or earlier. This professional group includes bricklayers, tile setters, builder’s laborers, concrete repairers, (stucco) plasterers, grouting workers, composition floor layers, terrazzo layers, floor fitters, parquet recliners, manufacturers of shaped bricks, manufacturers of concrete, and manufacturers of burnt lime products. To describe the spectrum of sensitization, we used data from the years 2009 – 2011. To analyze significant changes in the frequency of sensitization to certain allergens, we additionally evaluated data from the years 1994 – 2008, some of which have already been published [13]. 

IVDK is a network of currently 56 Departments of Dermatology in Germany, Switzerland, and Austria focusing on the epidemiological monitoring of contact dermatitis. All IVDK members are also members of the German Contact Dermatitis Research Group (Deutschen Kontaktallergie-Gruppe – DKG). A detailed description of the methods and organization of IVDK has been published elsewhere [26]. In summary: The participating centers assess clinical and patient data as well as the results of the patch tests of all tested patients in a standardized way and record them electronically. Every 6 months, the data are sent to the IVDK headquarters at the University of Göttingen where they are checked for quality and are subsequently entered into the IVDK’s central database [26, 31]. Processing and evaluation of the data is carried out according to published scientific standards [33] with the software SAS® (SAS Institute, Cary, NC, USA), current version 9.3. 

Patch tests were carried out and read according to the DKG guidelines [25]. Test results of day 3 were used for evaluation; only in very few cases where the reading was carried out on day 4 instead of day 3, the day-4 results were used. Most test preparations were obtained from Almirall Hermal, Reinbek, Germany. Some test preparations that were not available from Almirall were purchased from Chemotechnique, Vellinge, Sweden. The exposure time for the patch test was 48 hours (in 31,827 = 84.9% of the 37,487 patients tested between 2009 and 2011) or 24 hours (in 5,660 = 15.1% of patients), respectively. In 92.6% of patients, the test tape Finn-Chambers-on-Scanpor (8 mm inner chamber diameter) was used. 

## Results 

### Patients 

In all the dermatology departments that work together with the IVDK, patch tests were carried out in a total of 37,487 patients between 2009 and 2011. Of these, 415 (1.1%) belonged to the profession group “bricklayers and similar, manufacturer of construction material” (current or former profession). Of these 415 patients, 245 (59.0%) were tested because of occupation-related skin disease. The data of these 245 patients provided the basis for our evaluation of the current spectrum of sensitization. 

All patients were male; 159 patients (64.9%) were aged 40 years or older. Atopic dermatitis was present in 61 patients (24.9%). 167 patients (68.2%) suffered from hand eczema, 21 (8.6%) from facial eczema, and 11 (4.5%) from eczema on the legs. The patients’ current professions were as follows: 72 bricklayers (29.4%), 35 tile setters (14.3%), 30 builder’s laborers (12.2%), 18 concrete workers (7.3%), 18 floor fitters (7.3%), 8 (stucco) plasterers (3.3%), 6 parquet recliners (2.4%), 4 concrete repairers (1.6%), 3 composition floor/terrazzo layers (1.2%), 1 manufacturer of concrete stone (0.4%), and 1 grouting worker (0.4%). In 8 cases (3.3%), only the superordinate job title was indicated. The most frequently suspected allergen sources were (multiple answers were possible): construction material (161 patients (65.7%)), gloves (56 patients (22.9%)), adhesives (43 patients (17.6%)), and rubber (29 patients (11.8%)). The most frequent diagnoses were: allergic contact eczema (88 patients (35.9%)), chronic irritant contact eczema (46 patients (18.8%)), atopic eczema (28 patients (11.4%)), hyperkeratotic eczema (18 patients (7.3%)), dyshidrotic eczema (13 patients (5.3%)), nonclassified eczema (10 patients (4.1%)), acute irritant eczema (5 patients (2.0%)), and airborne contact eczema (5 patients (2.0%)). 

### Current spectrum of sensitization

****Most frequent allergens****

All allergens to which 5 patients or more (> 2% of the studied group) showed a positive reaction are listed in Table 1. The most frequent allergen was potassium dichromate (positive reactions in 15.1% of patients); the second most frequent allergen was epoxy resin (in 13.7%). Although, unlike the allergens of the DKG baseline series, they were not tested in 205 – 212 patients (83.7 – 86.5% of 245) but only in 159 – 181 patients (64.9 – 73.9% of 245), 8 further components of epoxy resin systems are among the 30 most frequent allergens – 5 reactive diluents (glycidyl ether) and 3 amine hardeners. Rubber ingredients that are not included in the DKG baseline series were tested in 132 – 136 patients (53.9 – 55.5% of 245) using patch testing; of these substances and of those rubber allergens included in the DKG baseline series, 9 are among the 30 most frequent allergens. 

****Test results using DKG test series****

Since 1999, the DKG task force “Occupational Test Series” has recommended carrying out patch tests when occupational contact allergy is suspected in bricklayers, construction workers, tile setters, and people working in similar jobs using the following DKG test series: baseline series, rubber series, synthetic resins/adhesives, building trade, ingredients of external agents, preservative agents (e.g., in external agents) [12]. Back then, the DKG test series “building trade” also included several allergens of other above-mentioned DKG test series. To avoid double testing, the test substances were reduced to 5 at the end of 2001; these 5 substances do not belong to any other test series recommended by the DKG. The evaluation of IVDK data obtained in 2006 – 2008 demonstrated that it is not necessary to test with all recommended DKG series because several test substances did not lead to reactions. Since September 2010, the DKG has recommended carrying out patch tests with the new DKG test series “building trade” and the DKG rubber series when occupational contact dermatitis is suspected in bricklayers, tile setters, etc. [11]. Patch testing using the DKG baseline series was carried out in 224 patients (91.4% of 245), the DKG test series “building trade” was used in 145 patients (59.2%), and the DKG rubber series was used in 135 patients (55.1%). Furthermore, the DKG test series “preservative agents, e.g., in external agents” was used in 167 patients (68.2%), the DKG test series “ingredients of external agents” was used in 164 patients (66.9%), and the DKG test series “synthetic resins/adhesives” was used in 138 patients (56.3%). The DKG test series “building trade” comprises 17 substances, 15 of which are also included in the DKG test series “synthetic resins/adhesives” (22 substances), among them all 12 epoxy resin allergens. These two DKG test series were tested in parallel in 88 patients. Due to the resulting overlap, data evaluation focused on the individual test substances irrespective of the test series the substance was tested in.

- DKG baseline series: The test reactions obtained using the DKG baseline series are presented in Table 2. Irritant control sodium lauryl sulfate 0.25% aqu. was tested in 207 patients. 162 patients (78.3%) showed no reaction (sls0). 35 patients had a mild irritant reaction (sls1), 9 patients had a more severe reaction (sls2), and 1 patient had a pronounced reaction (sls4).
- DKG test series “building trade”: The test results using the substances of the DKG test series “building trade” are listed in Table 3. Positive results were most frequently obtained with epoxy resin allergens, and among those in particular with 1,6-hexandiol diglycidyl ether (1,6-HDDGE) and 1,4-butanediol diglycidyl ether (1,4-BDDGE) as well as with m-xylidenediamine (MXDA). The 3 other allergens of synthetic resins that are not epoxy resin systems were virtually irrelevant. No positive reactions were observed using trimethylolpropane triglycidyl ether, a reactive diluent that is only rarely used in the building trade. Of the two preservative agents in the test series, methylisothiazolinone (MI) provoked more than twice as many positive reactions (6.5%) than benzisothiazolinone (BIT) (3.0% positive reactions).
- DKG rubber series: The test results using the DKG rubber series are presented in Table 4. The most frequent positive results were obtained with thiurams, followed by mercaptobenzothiazole derivatives and 1,3-diphenylguanidine. One single-positive reaction was observed with monobenzone and zinc dibutyldithiocarbamate, respectively. All other test substances of the rubber series did not provoke a positive reaction.
- DKG test series “preservative agents, e.g., in external agents”: The test results using the DKG test series “preservative agents, e.g., in external agents” are shown in Table 5. MI is the most frequent allergen in preservative agents (Table 1) and thus also the most important allergen of this test series. Furthermore, 2 patients showed positive reactions to iodpropinyl butylcarbamate and sodium benzoate, respectively; 1 patient had a positive reaction to sorbic acid. All other allergens did not cause positive test reactions.
- DKG test series “ingredients of external agents”: Table 6 shows the test results of the DKG test series “ingredients of external agents”. There were positive reactions to some allergens of this test series; however, there are usually even more questionable and/or irritant reactions that justify the characterization “problematic allergens” with a negative reaction index [6, 18]. Therefore, it has to be assumed that not all positive reactions to these allergens are indeed markers of sensitization.
- DKG test series “synthetic resins/adhesives”: The DKG test series “synthetic resins/adhesives” comprises 22 substances, 15 of which are also included in the DKG test series “building trade”. The reactions to these test substances are listed in Table 3. The other 7 test substances are: methacrylates, namely methyl methacrylate, 2-hydroxypropyl methacrylate (HEMA), 2-hydroxyethyl methacrylate (HPMA), ethylene glycol dimethacrylate (EGDMA), and triethylene glycol dimethacrylate (TEGDMA), BIS-GMA, and benzoyl peroxide. Only 2 patients reacted to the methacrylates: 1 patient reacted positively to all 5 methacrylates (except for BIS-GMA) and hydroxyethyl acrylate, 1 patient had a positive reaction to HEMA. 69 patients showed positive reactions to BIS-GMA. Benzoyl peroxide led to 4 positive reactions (3 +; 1 ++) as well as to 4 irritant and 9 questionable reactions, confirming that this test preparation was also a “problematic allergen”.

**Reaction coupling**

Among the frequent allergens, there are some for which reaction coupling by exposure coupling or by immunologic cross-reactions is known to exist. Some combinations are listed in Table 7. It can be seen that allergic reactions to cobalt chloride are more frequently coupled with reactions to potassium chromate (12 of 16 reactions) than with reactions to nickel sulfate (2 of 18 reactions). Positive reactions to methylisothiazolinone (MI) were highly associated with reactions to methylchloroisothiazolinone/methylisothiazolinone (MIC/MI) mix. About half of the allergic reactions to phenyl glycidyl ether (PGE) occurred concomitantly with reactions to epoxy resin. Simultaneous reactions to PGE and p-tert-butylphenyl glycidyl ether were observed in 4 of 10 cases. Both patients who showed positive reactions to cresyl glycidyl ether also reacted positively to PGE (not listed in Table 7). Coupling of allergic reactions to 1,6-hexanediol diglycidyl ether (1,6-HDDGE) and 1,4-butanediol diglycidyl ether (1,4-BDDGE) was high: Of 20 reactions to 1,6-HDDGE and 16 reactions to 1,4-BDDGE, 14 occurred concomitantly. 

Of the 29 patients with allergic reactions to epoxy resin, 20 reacted to further components of epoxy resin systems: 13 to MXDA, 11 to 1,6-HDDGE, and 10 to 1,4-BDDGE. All 6 patients who had a positive reaction to BIS-GMA also reacted positively to epoxy resin, but only 1 of these 6 patients reacted to 2-hydroxyethyl methacrylate (HEMA). 

Four of the 10 patients who showed a positive reaction to 4,4’-methylenedianiline (4,4’-MDA) also reacted to epoxy resin, and 3 also reacted to further components of epoxy resin systems, e.g., hardeners and/or reactive diluents. Another patient who had a positive reaction to 4,4’-MDA additionally reacted to isophorone diamine but not to other components of epoxy resin systems. 

In total, 28 patients reacted to thiurams, i.e., thiuram mix and/or at least one component of this mixture. Eight patients had positive reactions to mercaptobenzothiazole (MBT) and/or mercapto mix and/or at least one component of this mixture. Two patients showed positive reactions to zinc diethyldithiocarbamate (ZDEC) and 1 of these also to zinc dibutyldithioarbamate (ZDBC). For zinc dibenzylditiocarbmate, no positive tests were obtained. The 2 dithiocarbamate-positive patients also reacted to thiurams. Of the 8 patients who showed allergic reactions to MBT and/or MBT derivatives, 4 also reacted to thiurams. There were, however, no concomitant reactions to dithiocarbamates and MBT or MBT derivatives. 

### Chronology 

**Figure 1.**
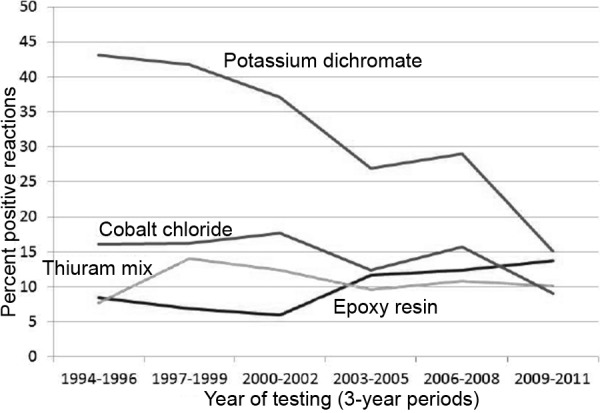
Frequency of allergic reactions to 4 occupational allergens. For interpretation please see text.

Figure 1 shows previously published [13] sensitization frequencies for the 4 most frequent occupational allergens, which were tested in more than 80% of the test group, plotted over time. These are: potassium dichromate, epoxy resin, thiuram mix, and cobalt chloride. The figure shows 3-year periods between 1994 and 2011. The frequency of sensitization to dichromate decreased from 43.1% in the period 1994 – 1996 to 15.1% in the period 2009 – 2011. Sensitization to cobalt also decreased: from 16.1% in 1994 – 1996 to 9.0% in 2009 – 2011. In contrast, sensitization to epoxy resin increased from 8.4% (1994 – 1997) to 13.7% (2009 – 2011). For thiuram mix, the frequency of positive reactions increased from 7.7% (1994 – 1996) to 10.1% (2009 – 2011). None of the courses was monotonic or linear. 

## Discussion 

The current spectrum of sensitization in bricklayers, tile setters, construction workers, and workers in similar professions who suffer from occupational dermatitis is dominated by dichromate, components of epoxy resin systems, and rubber ingredients. 

Although potassium dichromate is still the most frequent contact allergen, the declining trend of chromate sensitization in this group of patients, which was observed in the past years [13], has been continuing. Preventive measures to reduce allergic cement eczema, in particular the limitation of the chromate content in manually-processed cement to below 2 ppm, are obviously effective. That this measure could be effective in principle had already been known for more than 20 years in Scandinavian countries [4, 5, 24]. However, in Germany, no declining trend could be observed until 2000, and Bock et al. [36] had reported that in 335 occupational skin diseases diagnosed in the building trade according to the German Ordinance on Occupational Diseases (No. BK 5101), sensitizations to potassium dichromate were observed in 162 patients (48.4%), to cobalt dichloride in 67 (20%), to epoxy resins in 43 (12.8%), and to thiurams in 22 (6.6%). It was not until the year 2003 that an EU regulation stipulated the use of chromate-reduced cement when manually processed. Not only in Germany [13] but also in other European countries, this led to a decline in chromate sensitization in bricklayers who had occupational skin diseases [28, 29]. 

More or less in parallel with this decrease of chromate sensitizations, a decline of sensitizations to cobalt could be observed in the same patient group. It is well known that cobalt in cement frequently leads to cosensitization by coexposure in patients with allergic cement eczema due to chromate allergy [32]. In our patient collective, as well, allergic reactions to cobalt predominantly occurred in patients who were sensitized to chromate. Based on investigations by Fregert and Gruvberger [8] at the end of the 1970s, it has been assumed that an important requirement for the development of cobalt sensitization due to contact with cement is the presence of free amino acids in eczematous skin lesions, because under these circumstances water-soluble cobalt-amino acid complexes are built, which can cause sensitization. As the irritant properties of cement, and thus its ability to trigger irritant contact eczema, are not changed by reducing the chromate content, it could have been assumed that cobalt becomes the most important allergen in cement when chromate is reduced. In other words, when the frequency of irritant cement eczema does not decline and the presence of free amino acids in eczematous skin alone promotes cobalt sensitization, it could be assumed that the frequency of cobalt sensitization does not decrease in parallel with chromate sensitization. However, IVDK data do show such parallels (with certain reservations). This could have two possible explanations: Either there are factors other than the presence of free amino acids in eczematous skin lesions due to which cobalt sensitization develops (these could, for instance, be immunological processes in allergic contact eczema that result from primary sensitization to chromate), or the preventive measures, which were promoted simultaneously with the introduction of chromate-reduced cement, like, for example, the use of gloves when handling cement or cement-containing products, have led to a reduction of irritant cement eczema and thus contributed to the decreased frequency of contact allergy to cobalt. No data on the incidence of irritant cement eczema in Germany were available when this article was compiled. But if it had really decreased, one would have assumed that less bricklayers, construction workers, tile setters, and workers in similar jobs have registered with the IVDK for reasons of occupation-related eczema. This is, however, not the case. In the period 2000 – 2008, the IVDK registered 180 – 210 patients for each 3-year period [13]; in the period 2009 – 2011, the registered number was 245. This increase could possibly be traced back to the fact that more and more patients with allergic contact eczema caused by epoxy resin systems are being tested. Furthermore, the number of suspected occupation-related skin disease has markedly increased, which is surely related to the prevention campaign carried out in 2007/2008. While in the years 2003 and 2004, respectively, about 15,000 reports according to the German Ordinance on Occupational Diseases (No. BK 5101) were received, this number was approximately 18,500 in 2008, reaching circa 23,600 in 2010 [7]. Thus, our data cannot clearly answer why exactly there has been a simultaneous decrease in the incidences of chromate and cobalt sensitization in bricklayers, tile setters, construction workers, and workers in similar jobs. 

In general, nickel and cobalt sensitizations are highly associated, which is assumed to result from the presence of cobalt in nickel alloys, e.g., in costume jewelry [30]. Thus, the pronounced dissociation of sensitizations to nickel and cobalt in the group investigated by us is striking. Nickel is also present in cement, but not in a form where it could cause sensitization. Bricklayers and tile setters could have contact to nickel in handles of tools or the like [5, 8, 14, 27]. However, nickel allergy does not seem to play an important role from a quantitative point of view; the rate of sensitization is not higher than in men working in other professions [17]. The dissociation of nickel and cobalt sensitizations shows that cobalt sensitization was acquired by the handling of cement rather than by cobalt in nickel alloys. 

As has been described for previous years [13], sensitization to epoxy resin continues to increase in bricklayers, tile setters, and workers in similar jobs suffering from occupational dermatitis. In the meantime, the sensitization rate is almost as high as the rate of sensitization to potassium dichromate. Here, efforts of prevention seem to have not been very effective thus far. Therefore, there will have to be greater efforts in the future to recommend truly adequate gloves, to improve occupational hygiene, and to avoid direct skin contact with epoxy resin systems on construction sites. This kind of sensitization does not only involve the resin itself (the substance used for patch tests contains a resin on the basis of bisphenol A diglycidyl ether) but also reactive diluents and hardeners. Among the reactive diluents, 1,6-hexanediol diglycidyl ether (1,6-HDDGE) is the most frequent allergen, mostly involving a parallel reaction to 1,4-butanediol diglycidyl ether (1,4-BDDGE), which is significantly less prevalent than 1,6-HDDGE in epoxy resin products used in the construction business [19]. The concomitant reactions are certainly caused by immunologic cross-reactions to these chemically highly-related reactive diluents. The rate of positive reactions to phenyl glycidyl ether (PGE), which is no longer used in epoxy resin systems in the building trade, is remarkably high [19]. It is well known that patients who have a primary sensitization to epoxy resin on the basis of bisphenol A diglycidyl ether can also develop a cross-reaction to PGE [22, 23]. This might explain some of the reactions (5 of 9 positive reactions to PGE). However, there are also 4 patients in the group investigated by us who had an allergic reaction to PGE but not to epoxy resin. It is unclear whether in these cases a primary sensitization to PGE is indeed present or if the sensitization to PGE is instead caused by an immunologic cross-reaction, while a primary sensitization to other aromatic glycidyl ethers, e.g., cresyl glycilyl ether (CGE) or p-tert-butylphenyl glycidil ether (PTBPGE) is present. Both are still being used in epoxy resin systems in the building trade, albeit not very extensively [19]. Of note, trimethylolpropane triglycidyl ether (TMPTGE) did not cause any allergic reaction. According to the information system of hazardous materials published by the German Institution for Statutory Accident Insurance and Prevention in the building trade (GISBAU), these reactive diluents are only rarely present in epoxy resin systems used in the building trade [19]. It remains unclear whether its low sensitizing potency or the fact that it is only rarely used is the reason why no sensitization to TMPTGE was observed in our patient collective. 

Among the hardeners used in epoxy resin systems, m-xylidenediamine (MXDA) is by far the most frequent allergen. Sensitization has been observed in approximately 3-times as many cases as sensitization to isophorone diamine (IPDA). Both amine hardeners are extensively used in epoxy resin systems in the building trade [19]. 

4,4’-methylenedianiline (4,4’-MDA) has not been used in epoxy resin systems for years [19]. According to current investigations, it has been assumed that positive reactions to 4,4’-MDA are also a marker for sensitization to 4,4’-diphenylmethane diisocyanate (4,4’-MDI) because 4,4’-MDA is the corresponding amine of this diisocyanate [1]. Diisocyanates are basic chemical components of polyurethane, which is used as fitting or insulating foam or as a glue. Thus, people working on construction sites could be exposed to 4,4’-MDI. The problem with using diisyocyanates for skin testing is that these test substances frequently do not contain the indicated diisocyanate concentration because of their high reactivity; thus, they do not reliably reflect sensitization [9]. Occupational dermatologists in Scandinavia are therefore recommending patch testing with 4,4’-MDA to detect sensitization to 4,4’-MDI [1, 10]. The fact that 4 of our 10 patients who reacted to 4,4’-MDA also reacted positively to epoxy resin, and that 3 of them were also sensitized to further components of epoxy resin systems, probably reflects coexposure to polyurethane products and epoxy resin systems. Immunologic cross-reactions between 4,4’-MDA, on the one hand, and epoxy resins, reactive diluents, or other amine hardeners (like MXDA or IPDA), on the other, are not to be assumed, nor is exposure to 4,4’-MDA due to epoxy resin systems. 

The proportion of allergic reactions to thiurams (28 patients (11.4%) of our patient collective) is strikingly high. In general, thiurams are still the most frequent allergens in rubber gloves [15]. It can be assumed that in our collective, rubber gloves were an important source of sensitization to thiurams. Further allergen sources could be other things made of rubber, like boots, handles, and so forth. Dithiocarbamates, which are chemically related to thiurams, led to allergic reactions in only 2 patients. Both of them were allergic to thiurams. It can be hypothesized that in these patients, immunologic cross-reactions between thiurams and dithiocarbamates are present, although exposure coupling cannot be excluded. Sensitization to mercaptobenzothiazole and its derivatives was observed significantly less often than sensitization to thiurams. In the light of this, thiuram-free rubber gloves should be recommended for the building trade, as has been done for other sectors [21, 35]. 

As mentioned above, since September 2010, the DKG has recommended carrying out patch tests with the new DKG test series “building trade” and the DKG rubber series along with the DKG standard series when occupational contact dermatitis is suspected in bricklayers, tile setters, etc. [11]. As our results show, these tests cover the allergens that are most relevant for this patient group. The most important allergen in preservative agents, methylisothiazolinone (MI), is not only included in the DKG test series “preservative agents, e.g., in external agents”, but also in the DKG test series “building trade”. There was an almost complete accordance of positive reactions to MI and the methylchloroisothiazolinone/methylisothiazolinone (MCI/MI) mix, which is included in the DKG standard series. A recent detailed analysis of IVDK data suggests that primary sensitization to MI has increased over the last years; immunologic cross-reaction then leads to allergic reactions to MCI (and thus to the test formulation MCI/MI) [16]. MI is also present in various materials used in the building trade so that the sensitization is of occupational relevance in many cases. 

The DKG test series “ingredients of external agents” and “preservative agents, e.g., in external agents”, which were frequently carried out as additional tests in our patient collective, only rarely provided further relevant results. Using the preservative agents series, reactions to iodopropynyl butylcarbamate, sodium benzoate, and sorbic acid were observed sporadically, while all other allergens did not provoke any positive reaction. 

Using the DKG test series “ingredients of external agents” several positive reactions were observed; however, also questionable and irritant reactions were obtained for the allergens contained in this series, resulting in an unfavorable reaction index [6]. It has to be assumed that not all reactions observed with this test series were indeed type IV sensitizations. In fact, some of the reactions could well have been false positive, in particular if they were only single positive [18]. Given the low yield of unambiguous allergic reactions, it has to be questioned whether this DKG test series should be routinely used in bricklayers, tile setters, and workers in similar jobs in which occupational contact eczema is suspected. In our opinion, well-targeted testing when intolerance of external agents or ointment bases is suspected seems to be more sensible. Moreover, external agents provided by the patient can easily be tested, and in the case of a positive reaction, the causative agent can be detected by further well-targeted tests [20]. 

The following conclusions can be drawn from our data analysis:

- The exclusive use of chromate-reduced cement for manual processing, which has been mandatory by EU regulation since 2003, continues to be successful; sensitization to chromate in bricklayers, construction workers, and tile setters with occupational dermatitis continues to decrease.
- Sensitization to epoxy resins and further components of epoxy resin systems, like reactive diluents and amine hardeners, are still of increasing importance in this occupational field. More intensive preventive measures, in particular improved work hygiene to avoid direct skin contact, are required.
- Thiurams are of major importance as allergens in bricklayers, construction workers, tile setters, and workers in similar jobs, who suffer from occupational dermatitis. When recommending gloves for this business sector, thiuram-free products should be preferred.
- The DKG recommendation that has been valid since September 2010 and recommends the use of the DKG standard series, the DKG test series “building trade”, and the DKG rubber series when occupational contact allergy is suspected in bricklayers, construction workers, tile setters, and workers in similar jobs, is still correct. These test series cover all allergens important in this sector. More extensive tests are not necessary for routine testing; further tests should only be carried out if necessary judging on individual patient history.

## Acknowledgment 

The following IVDK centers contributed data for analysis (in alphabetic order; names of responsible physicians in parentheses): Augsburg (A. Ludwig), Basel (A. Bircher), Berlin Charité (M. Worm), Bern (D. Simon), Bielefeld (I. Effendy), Bochum IPA (M. Fartasch), Dortmund (P.J. Frosch, K. Kügler), Dresden (P. Spornraft-Ragaller, A. Bauer), Dresden Friedrichstadt (A. Koch), Erlangen (V. Mahler), Essen (U. Hillen), Falkenstein (H. Schwantes), Freudenberg (C. Szliska), Gera (J. Meyer, H. Grunwald-Delitz, M. Kaatz), Graz (B. Kränke, W. Aberer), Göttingen (T. Fuchs, J. Geier), Halle (B. Kreft), Hamburg (E. Coors), Hamburg BUK (K. Breuer, U. Seemann, C. Schröder-Kraft), Hannover (T. Schaefer, T. Werfel), Heidelberg (M. Hartmann, K. Schäkel), Heidelberg AKS (T.L. Diepgen, E. Weisshaar), Homburg/Saar (C. Pföhler), Jena (S. Schliemann), Kiel (J. Brasch), Leipzig (R. Treudler), Lippe Detmold (S. Nestoris), Lübeck (J. Grabbe, I. Shimanovich), Mainz (D. Becker), Mannheim (D. Booken, C.-D. Klemke), Minden (R. Stadler), Munich LMU (B. Przybilla, P. Thomas, R. Eben), Munich Schwabing (M. Agathos, G. Isbary, K. Ramrath), Munich TU (U. Darsow), Münster (B. Hellweg, R. Brehler), Nuremberg (A. Bachtler), Osnabrück (C. Skudlik, S.M. John), Rostock (J. Trcka), Tübingen (T. Biedermann, J. Fischer), Würzburg (A. Trautmann), Zwickau (D. Teubner), Zurich (B. Ballmer-Weber). 

## Conflict of interest 

The authors have no conflict of interest. 

**Table 1.** IVDK, 2009 – 2011: the 30 most frequent allergens in 245 bricklayers, tile setters, etc. with occupational dermatitis. Percentage of reactions and exact 95% confidence interval (95% CI).

Test substance	Tested patients	Patients showing positive reaction	% positive (95% CI)
Potassium dichromate	205	31	15.1 (10.5 – 20.8)
Epoxy resin	212	29	13.7 (9.4 – 19.1)
Thiuram mix	208	21	10.1 (6.4 – 15.0)
1,6-Hexandiol diglycidyl ether	163	20	12.3 (7.7 – 18.3)
Cobalt chloride	210	19	9.0 (5.5 – 13.8)
m-xylidenediamine	159	18	11.3 (6.8 – 17.3)
Tetraethylthiuram disulfide	133	17	12.8 (7.6 – 19.7)
Tetramethylthiuram monosulfide	132	16	12.1 (7.1 – 18.9)
1,4-butanediol diglycidyl ether	163	16	9.8 (5.7 – 15.5)
Balsam of Peru	212	12	5.7 (3.0 – 9.7)
Phenyl glycidyl ether	181	12	6.6 (3.5 – 11.3)
Tetramethylthiuram disulfide	133	12	9.0 (4.7 – 15.2)
Methylisothiazolinone	170	11	6.5 (3.3 – 11.3)
4,4’-Methylenedianiline	182	10	5.5 (2.7 – 9.9)
Nickel sulphate	210	10	4.8 (2.3 – 8.6)
p-tert-butylphenyl glycidyl ether	159	10	6.3 (3.1 – 11.3)
(Chloro-)Methylisothiazolinone (MIC/MI)	210	9	4.3 (2.0 – 8.0)
Colophonium	212	8	3.8 (1.6 – 7.3)
Butyl glycidyl ether	163	8	4.9 (2.1 – 9.4)
Dipentamethylenethiuram disulfide	134	6	4.5 (1.7 – 9.5)
Fragrance mix	209	6	2.9 (1.1 – 6.1)
Isophorone diamine	164	6	3.7 (1.4 – 7.8)
Morpholinyl mercaptobenzothiazole	136	6	4.4 (1.6 – 9.4)
BIS-GMA	138	6	4.3 (1.6 – 9.2)
Mercapto mix	211	6	2.8 (1.1 – 6.1)
Methyldibromo glutaronitrile	211	5	2.4 (0.8 – 5.4)
Mercaptobenzothiazole (MBT)	211	5	2.4 (0.8 – 5.4)
N-Isopropyl-N’-phenyl-p-phenylenediamine	209	5	2.4 (0.8 - 5.5)
1,2-Benzisothiazolin-3-one, sodium salt	164	5	3.0 (1.0 – 7.0)
Trimethylhexane-1,6-diamine	159	5	3.1 (1.0 – 7.2)

**Table 2.** Test results using the DKG standard series. Right column shows percentage of positive reactions (% pos.).

Substance	Conc.	Unit	Base	No. of tests	neg.	?	q	+	++	+++	ir.	% pos.
Potassium dichromate	0.50	%	pet.	205	165	7	1	21	7	3	1	15.1
Epoxy resin	1.00	%	pet.	212	180	2	0	14	12	3	1	13.7
Thiuram mix	1.00	%	pet.	208	184	2	0	7	9	5	1	10.1
Cobalt chloride	1.00	%	pet.	210	187	3	0	11	7	1	1	9.0
Balsam of Peru	25.00	%	pet.	212	190	3	0	11	1	0	7	5.7
Nickel sulphate	5.00	%	pet.	210	199	0	0	3	5	2	1	4.8
(Chloro-)Methylisothiazolinone (MIC/MI)	100.00	ppm	aqu.	210	200	1	0	6	3	0	0	4.3
Colophonium	20.00	%	pet.	212	203	1	0	4	3	1	0	3.8
Fragrance mix	8.00	%	pet.	209	196	3	0	5	1	0	4	2.9
Mercapto mix without MBT	1.00	%	pet.	211	204	1	0	4	0	2	0	2.8
Methyldibromo glutaronitrile (dibromodicyanobutane)	0.20	%	pet.	202	188	6	1	4	0	1	2	2.5
N-Isopropyl-N’-phenyl-p-phenylenediamine	0.10	%	pet.	209	202	1	1	1	2	2	0	2.4
Mercaptobenzothiazole (MBT)	2.00	%	pet.	211	204	2	0	3	2	0	0	2.4
Composits mix	5.00	%	pet.	202	195	4	0	2	1	0	0	1.5
Fragrance mix II	14.00	%	pet.	210	202	3	0	2	1	0	2	1.4
Lanolin alcohols	30.00	%	pet.	211	206	2	0	1	2	0	0	1.4
Propolis	10.00	%	pet.	210	201	5	1	1	1	0	1	1.0
Cetostearyl alcohol	20.00	%	pet.	211	203	4	0	1	1	0	2	0.9
Zinc diethyldithiocarbamate	1.00	%	pet.	212	205	4	1	1	0	1	0	0.9
Ylang-ylang (I + II) oil*	10.00	%	pet.	133	130	2	0	1	0	0	0	0.8
Jasmine absolute*	5.00	%	pet.	133	130	1	0	1	0	0	1	0.8
Sandalwood oil*	10.00	%	pet.	133	132	0	0	1	0	0	0	0.8
Formaldehyde	1.00	%	aqu.	211	206	2	1	0	1	0	1	0.5
Paraben mix	16.00	%	pet.	211	204	5	0	1	0	0	1	0.5
Turpentine	10.00	%	pet.	211	206	4	0	1	0	0	0	0.5
Hydroxyisohexyl 3-cyclohexene carboxaldehyde (HICC; Lyral®)	5.00	%	pet.	212	209	2	0	0	1	0	0	0.5
Bufexamac	5.00	%	pet.	209	207	2	0	0	0	0	0	0.0
p-tert-Butylphenol formaldehyde resin	1.00	%	pet.	212	211	1	0	0	0	0	0	0.0
Bronopol	0.50	%	pet.	211	207	2	0	0	0	0	2	0.0

*Lower number of tests because the test formulation was not included in the DKG standard series before September 2010.

**Table 3.** Test results using the test substances included in the DKG test series “building trade”. Right column shows percentage of positive reactions (% pos.).

Substance	Conc.	Unit	Base	No. of tests	neg.	?	q	+	++	+++	ir.	% pos.
1,6-hexanediol diglycidyl ether	0.25	%	pet.	163	139	2	1	11	5	4	1	12.3
m-xylidenediamine	0.10	%	pet.	159	141	0	0	10	7	1	0	11.3
1,4-butanediol diglycidyl ether	0.25	%	pet.	163	135	6	1	10	3	3	5	9.8
Phenyl glycidyl ether	0.25	%	pet.	181	161	7	1	9	2	1	0	6.6
Methylisothiazolinone	0.05	%	aqu.	170	157	2	0	6	4	1	0	6.5
p-tert-butylphenyl glycidyl ether	0.25	%	pet.	159	146	1	2	6	4	0	0	6.3
4,4’-Methylenedianiline	0.50	%	pet.	182	165	6	0	5	4	1	1	5.5
Butyl glycidyl ether	0.25	%	pet.	163	153	2	0	5	2	1	0	4.9
Isophorone diamine	0.50	%	pet.	164	156	1	1	4	1	1	0	3.7
trimethylhexane-1,6-diamine (isomer mixture)	0.50	%	pet.	159	150	1	1	2	3	0	2	3.1
1,2-Benzisothiazolin-3-one, sodium salt	0.10	%	pet.	164	154	3	0	5	0	0	2	3.0
Diethylenetriamine	1.00	%	pet.	160	157	1	0	1	1	0	0	1.3
Cresyl glycidyl ether	0.25	%	pet.	164	155	6	1	2	0	0	0	1.2
Hydroxyethyl acrylate	0.10	%	pet.	164	159	3	0	2	0	0	0	1.2
Phenol-formaldehyde resin (Novolak)	5.00	%	pet.	181	177	1	0	1	1	0	1	1.1
p-tert-butylcatechol	0.25	%	pet.	195	193	1	0	1	0	0	0	0.5
Trimethylolpropane triglycidyl ether	0.25	%	pet.	157	155	2	0	0	0	0	0	0.0

**Table 4.** Test results using the DKG rubber series. Right column shows percentage of positive reactions (% pos.).

Substance	Conc.	Unit	Base	No. of tests	neg.	?	q	+	++	+++	ir.	% pos.
Tetraethylthiuram disulfide (Disulfiram)	0.25	%	pet.	132	114	1	0	8	7	2	0	12.9
Tetramethylthiuram monosulfide	0.25	%	pet.	131	113	1	1	8	7	1	0	12.2
Tetramethylthiuram disulfide	0.25	%	pet.	132	119	1	0	5	6	1	0	9.1
Morpholinyl mercaptobenzothiazole	0.50	%	pet.	132	126	0	0	4	1	1	0	4.5
Dipentamethylenethiuram disulfide	0.25	%	pet.	133	125	1	0	5	1	0	1	4.5
1,3-Diphenylguanidine	1.00	%	pet.	132	125	2	0	3	1	0	1	3.0
N,N’-Diphenyl-p-phenylenediamine	0.25	%	pet.	132	128	0	0	1	3	0	0	3.0
N-Cyclohexyl-2-benzothiazole sulfenamide	1.00	%	pet.	132	128	0	0	2	1	1	0	3.0
Dibenzothiazyl disulfide	1.00	%	pet.	132	127	1	0	2	2	0	0	3.0
Monobenzone	1.00	%	pet.	132	128	3	0	1	0	0	0	0.8
Zinc dibutyldithiocarbamate	1.00	%	pet.	133	131	0	1	1	0	0	0	0.8
4,4‘-Dihydroxydiphenyl	0.10	%	pet.	132	132	0	0	0	0	0	0	0.0
Dibutylthiourea	1.00	%	pet.	132	132	0	0	0	0	0	0	0.0
Diphenylthiourea	1.00	%	pet.	132	132	0	0	0	0	0	0	0.0
Ethylenediamine-di-HCl	1.00	%	pet.	131	131	0	0	0	0	0	0	0.0
Methenamine (hexamethylenetetramine)	1.00	%	pet.	132	131	1	0	0	0	0	0	0.0
p-tert-butylcatechol	0.25	%	pet.	132	132	0	0	0	0	0	0	0.0
Cyclohexylthiophthalimide	0.50	%	pet.	132	132	0	0	0	0	0	0	0.0
Zinc dibenzyldithiocarbamate	1.00	%	pet.	133	133	0	0	0	0	0	0	0.0

**Table 5.** Test results using the DKG test series “preservative agents, e.g., in external agents”: Right column shows percentage of positive reactions (% pos.).

Substance	Conc.	Unit	Base	No. of tests	neg.	?	q	+	++	+++	ir.	% pos.
Methylisothiazolinone	0.05	%	aqu.	157	145	2	0	6	4	0	0	6.4
Iodopropynyl butylcarbamate	0.20	%	pet.	161	152	6	1	2	0	0	0	1.2
Sodium benzoate	5.00	%	pet.	163	158	2	0	2	0	0	1	1.2
Sorbic acid	2.00	%	pet.	160	156	2	0	0	1	0	1	0.6
Quaternium-15	1.00	%	pet.	160	160	0	0	0	0	0	0	0.0
Benzyl alcohol	1.00	%	pet.	160	160	0	0	0	0	0	0	0.0
Chloroacetamide	0.20	%	pet.	160	160	0	0	0	0	0	0	0.0
Chlorhexidine digluconate	0.50	%	aqu.	162	161	1	0	0	0	0	0	0.0
Diazolidinyl urea (Germall II)	2.00	%	pet.	160	160	0	0	0	0	0	0	0.0
Imidazolidinyl urea (Germall 115)	2.00	%	pet.	160	160	0	0	0	0	0	0	0.0
Triclosan	2.00	%	pet.	160	160	0	0	0	0	0	0	0.0
DMDM Hydantoin	2.00	%	aqu.	160	159	1	0	0	0	0	0	0.0

**Table 6.** Test results using the DKG test series “ingredients of external agents”. Right column shows percentage of positive reactions (% pos.).

Substance	Conc.	Unit	Base	No. of tests	neg.	?	q	+	++	+++	ir.	% pos.
Cocamidopropyl betaine	1.00	%	aqu.	161	148	3	0	4	0	0	6	2.5
Propylene glycol	20.00	%	aqu.	158	149	4	0	1	1	1	2	1.9
Amerchol L-101	50.00	%	pet.	159	154	1	0	2	1	0	1	1.9
Cetostearyl alcohol	20.00	%	pet.	152	146	3	0	1	1	0	1	1.3
Octyl gallate	0.30	%	pet.	161	139	13	0	2	0	0	7	1.2
Benzophenone-4 (Sulisobenzone)	10.00	%	pet.	152	149	2	0	1	0	0	0	0.7
Butylhydroxyanisole (BHA)	2.00	%	pet.	160	159	1	0	0	0	0	0	0.0
Butylated hydroxytoluene (BHT)	2.00	%	pet.	160	159	0	1	0	0	0	0	0.0
Polyethylene glycol ointment DAB 8	100.00	%		159	159	0	0	0	0	0	0	0.0
tert-Butylhydroquinone	1.00	%	pet.	161	157	2	0	0	0	0	2	0.0
Triethanolamine (TEA;Trolamine)	2.50	%	pet.	159	157	1	0	0	0	0	1	0.0
Cocamide diethanolamine	0.50	%	pet.	158	153	4	0	0	0	0	1	0.0

**Table 7.** Simultaneous reactions to various allergens.

pos.		Cobalt chloride		
		neg., quest., ir.	Sum	
Potassium dichromate	pos.	12	19	31
	neg., quest., ir.	4	169	173
	Sum	16	188	204
pos.		Cobalt chloride		
		neg., quest., ir.	Sum	
Nickel sulphate	pos.	2	8	10
	neg., quest., ir.	16	182	198
	Sum	18	190	208
pos.		Methylisothiazolinone		
		neg., quest., ir.	Sum	
Methylchloroisothiazolinone/ methylisothiazolinone	pos.	7	1	8
	neg., quest., ir.	2	146	148
	Sum	9	147	156
pos.		Phenyl glycidyl ether		
		neg., quest., ir.	Sum	
Epoxy resin	pos.	5	22	27
	neg., quest., ir.	4	133	137
	Sum	9	155	164
pos.		Phenyl glycidyl ether		
		neg., quest., ir.	Sum	
p-tert-butylphenyl glycidyl ether	pos.	4	6	10
	neg., quest., ir.	6	143	149
	Sum	10	149	159
pos.		1,4-butanediol diglycidyl ether		
		neg., quest., ir.	Sum	
1,6-hexanediol diglycidyl ether	pos.	14	6	20
	neg., quest., ir.	2	140	142
	Sum	16	146	162
